# Heparin-binding epidermal growth factor-like growth factor is a potent regulator of invasion activity in oral squamous cell carcinoma

**DOI:** 10.3892/or.2011.1616

**Published:** 2011-12-30

**Authors:** YUICHI OHNISHI, HIROSHI INOUE, MASAYUKI FURUKAWA, KENJI KAKUDO, MASAMI NOZAKI

**Affiliations:** 1 Second Department of Oral and Maxillofacial Surgery, Osaka Dental University, Chuo-ku, Osaka 540-0008; 2 Department of Cell Biology, Research Institute for Microbial Diseases, Osaka University, Suita, Osaka 565-0871, Japan

**Keywords:** oral cancer, squamous cell carcinoma, invasion, matrix metalloproteinases, heparin-binding epidermal growth factor-like growth factor, epidermal growth factor receptor, ectodomain shedding

## Abstract

Heparin-binding epidermal growth factor (EGF)-like growth factor (HB-EGF) has been shown to stimulate the growth of various cell types in an autocrine or paracrine manner. Although HB-EGF is widely expressed in tumors when compared with normal tissue, its contribution to cancer progression remains obscure. The objective of this study was to explore the effects of HB-EGF on proliferation, invasion activity and MMP-9 levels of an oral squamous cell carcinoma cell line, HSC3, *in vitro*. MTT assays, Matrigel invasion assays and RT-PCR in combination with RNA interference (RNAi) were used in this study. An RNAi-mediated decrease in HB-EGF expression reduced invasion activity and MMP-9 mRNA levels, but not proliferation, in HSC3 cells. The addition of purified HB-EGF to cell culture medium upregulated MMP-9 mRNA levels in HSC3 cells. Furthermore, the TACE inhibitor TAPI-2 or EGFR inhibitor AG1478 decreased MMP-9 mRNA levels in HSC3 cells. These data indicate that HB-EGF released from HSC3 cells by TACE stimulates EGFR in an autocrine manner, which in turn activates invasion activity via MMP-9 upregulation.

## Introduction

Squamous cell carcinoma (SCC) occurs most frequently in the oral and maxillofacial region, and its metastatic ability conveys a poor prognosis. The standard treatment for oral cancer is a combination of surgery, radiation, and chemotherapy. Better insight into the mechanisms of progression of this cancer, of which one major issue is metastasis, is clearly needed, and discovery of novel molecular targets to assist the development of new therapeutic strategies is vital. Metastasis is a multi-step process by which primary tumor cells invade adjacent tissues, enter the systemic circulation (intravasate), translocate through the vasculature, arrest in distant capillaries, extravasate into the surrounding tissue parenchyma, and finally proliferate from a tiny cell mass into large secondary tumors in a foreign environment (1). In the past decade, studies have been carried out to investigate the genes and gene products that drive the metastatic process. Many molecules have been identified, some of which are involved in primary tumor-specific and target tissue-specific manners (2,3). Determination of the molecules involved in oral SCC metastasis is necessary.

Signal transduction by the epidermal growth factor (EGF) family of ligands has been demonstrated to promote tumorigenesis and metastasis (4,5). Several studies using EGF receptor (EGFR) inhibitors have indicated that EGF/EGFR signaling mediates osteolytic bone metastasis of breast, prostate, and kidney cancers (6). Heparin-binding epidermal growth factor-like growth factor (HB-EGF) contributes to cell adhesion, invasion, and angiogenesis associated with transcoelomic metastasis in ovarian cancer (7). In addition, HB-EGF was identified as one of the mediators of cancer cell passage through the blood-brain barrier during metastasis (3). These findings suggest that HB-EGF is important in several metastatic processes.

HB-EGF is initially synthesized as a transmembrane protein, similar to other members of the EGF family. The membrane-anchored form of HB-EGF (pro-HB-EGF) is cleaved at the cell surface by a protease to yield the soluble form (s-HB-EGF); this process is known as ectodomain shedding (8,9). s-HB-EGF has potent mitogenic and chemoattractant activities for a number of cell types (10). In many cases, s-HB-EGF released from cancer cells is involved in oncogenic transformation, tumor invasion, and metastasis (11,12). Although it is interesting whether HB-EGF affects oral SCC metastasis, there is limited evidence supporting their relation.

The present study examined whether HB-EGF affects metastasis in oral SCC. The results indicate that when HB-EGF is overexpressed in oral SCC cells, s-HB-EGF is released by shedding and subsequently increases invasion activity through upregulation of MMP-9 downstream of EGFR, in an autocrine manner.

## Materials and methods

### Reagents

Recombinant human HB-EGF was purchased from Wako Pure Chemical Industries, Ltd. TAPI-2 and AG1478 were purchased from Calbiochem.

### Cell culture and RNA extraction

The human tongue squamous cell carcinoma cell line HSC3 was obtained from the Human Science Research Resource Bank (Osaka, Japan). HSC3 cells were grown as monolayers in Dulbecco’s modified Eagle’s medium (DMEM) supplemented with 10% fetal bovine serum (FBS) in humidified 5% CO_2_ in air at 37°C in a CO_2_ incubator (Sanyo, Japan). Total-RNA was isolated using a Qiagen RNeasy mini kit (Qiagen) or TRIzol reagent (Invitrogen, Carlsbad, CA), according to the manufacturer’s instructions.

### Real-time quantitative PCR and reverse transcription-PCR

First-strand cDNA synthesis was performed using 2 μg of total-RNA and TaqMan reverse transcription reagents (Roche) for real-time PCR, and using 1 μg of total-RNA and SuperScript III for reverse transcription-PCR, following the manufacturer’s instructions. The TaqMan quantitative PCR reaction was carried out using the following oligonucleotide primers: β-actin (forward 5′-AAACTGGACGGTGGAGGT-3′ and reverse 5′-AG AGAAGTGGGGTGGCTTTT-3′); amphiregulin (forward 5′-GA GAAGCTGAGGAACGAAAGAA-3′ and reverse 5′-AGGACC GACTCATCATTTATGG-3′); epigen (forward 5′-GCCCTATA ATGTGTCAGGCACT-3′ and reverse 5′-GAAGGCAAATTTT TACCACTCG-3′); epiregulin (forward 5′-GAGAAGGGGGA GTAATGACTTG-3′ and reverse 5′-AAGTGCAATTACAGA GTGCAAAA-3′); HB-EGF (forward 5′-GGAACTCACTTTC CCTTGTGTC-3′ and reverse 5′-CTCAGCCTTTTGCTTT GCTAAT-3′); TGF-α (forward 5′-GAAGGAGGAATGACTCA AATGC-3′ and reverse 5′-AAGCCTGGTAAATCAATGG CTA-3′); betacellulin (forward 5′-GAATGTGTCTCAGGAA AAACAGC-3′ and reverse 5′-TGTTGCTACCTAACCAGT TGCT-3′); EGF (forward 5′-TTGGGACAACAGTGCTTTG TAA-3′ and reverse 5′-CTGACCAAACCAGTGTGACTGT-3′). Experiments were performed independently in triplicate. To examine the expression of MMP-9, β-actin and GAPDH, reverse transcription-PCR analysis was carried out using primers specific for MMP9 (forward 5′-GTGCTCCTGGTGC TGGGCTG-3′ and reverse TCCAGCTCACCGGTCTCGGG); β-actin (forward 5′-GAAAATCTGGCACCACACCTT-3′ and reverse 5′-TTGAAGGTAGTTTCGTGGAT-3′); GAPDH (forward 5′-ACAGTCAGCCGCATCTTCTT-3′ and reverse 5′-TGGAAGATGGTGATGGGATT-3′).

### RNA interference

HSC3 cells were transfected with HB-EGF siRNA oligos (sense 5′-GGACCCAUGUCUUCGGAAA-3′ and antisense 5′-UUUCCGAAGACAUGGGUCC-3′) or control scrambled siRNA oligos (sense 5′-AUCCGCGCGAU AGUACGUA-3′ and antisense 5′-UACGUAGUAUCGCGCG GAU-3′) (B-Bridge International, Mountain View, CA) using Lipofectamine™ RNAiMAX (Invitrogen) according to the manufacturer’s instructions, followed by incubation for 48 h at 37°C in a humidified atmosphere of 5% CO_2_ in air.

### Cell proliferation assay

Cell proliferation was assessed using a 3-[4,5-dimethylthiazol-2-yl]-2,5-diphenyl tetrazolium bromide (MTT) assay at three time points, Days 1, 2 and 3 after seeding. MTT reagent (50 μl) was added to each well, and the plates were incubated for 1 h at 37°C. After the reaction, 500 μl of isopropanol containing 0.04 N HCl were added to each well. The reactions were transferred to a 96-well plate, and the absorbance was measured at a test wavelength of 570 nm and reference wavelength of 630 nm with a microplate reader (Model 680; Bio-Rad).

### In vitro cell invasion assay

Cell invasion assays were performed according to the manufacturer’s instructions. Briefly, cells (2×10^4^/well) were seeded in a 6-well BioCoat Matrigel Invasion Chamber (Becton-Dickinson, Bedford, MA) in DMEM containing 10% (v/v) heat-inactivated FBS. After 48 h of incubation, non-invading cells were removed from the upper surface of the membrane by scrubbing, and the membrane was stained using a Diff-Quik staining kit. Invading cells were counted under a microscope, and the invasion index (= cells migrating through Matrigel-coated membrane/cells migrating through control insert membrane) was determined.

### Zymogram

After cells had been incubated in serum-free medium for 48 h, the conditioned medium was collected for zymography. Samples were diluted in SDS-polyacrylamide gel electrophoresis (SDS-PAGE) sample buffer and electrophoresed in 12.5% polyacrylamide gels containing gelatin (0.1%), at 60 mA for 2 h at 4°C. The gels containing the separated sample proteins were incubated overnight in 0.05 M Tris/HCl (pH 7.5) containing 10 M CaCl_2_, then stained with Coomassie Blue (0.25%), and de-stained in methanol/acetic acid/water (50:10:40).

### Statistical analysis

The Mann-Whitney U test was used to assess the statistical significance of differences between samples. P-values <0.05 were considered to indicate statistical significance.

## Results

### HB-EGF is associated with the invasion potential of oral SCC HSC3 cells

**Figure 1:**
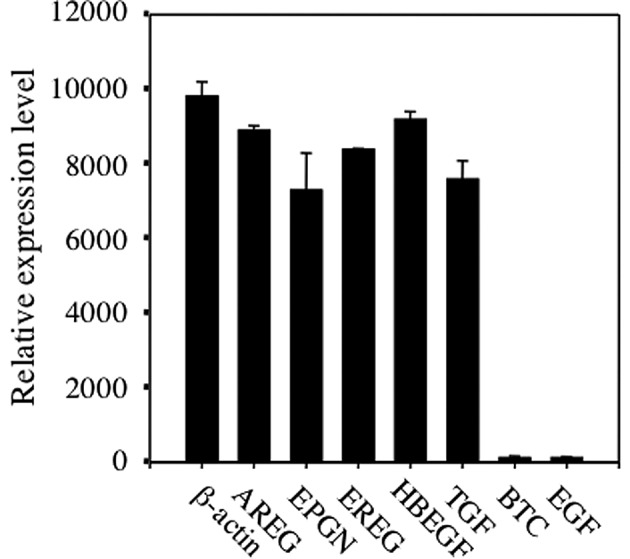
Expression levels of EGF family mRNA in HSC3 cells. Amphiregulin (AREG), epigen (EPHN), epiregulin (EREG), HB-EGF (HBEGF), TGF-α (TGF), betacellulin (BTC), EGF and β-actin mRNA expression levels in HSC3 cells were analyzed by RT-qPCR.

Expression of EGF family mRNA in HSC3 cells was analyzed by real-time RT-PCR. The expression levels of HB-EGF were high in HSC3 cells (Fig. 1). In addition, high expression levels of amphiregulin (AREG), epigen (EPGN), epiregulin (EREG), and TGF-α mRNA were detected, but betacellulin (BTC) and EGF mRNA were not highly expressed (Fig. 1).

**Figure 2:**
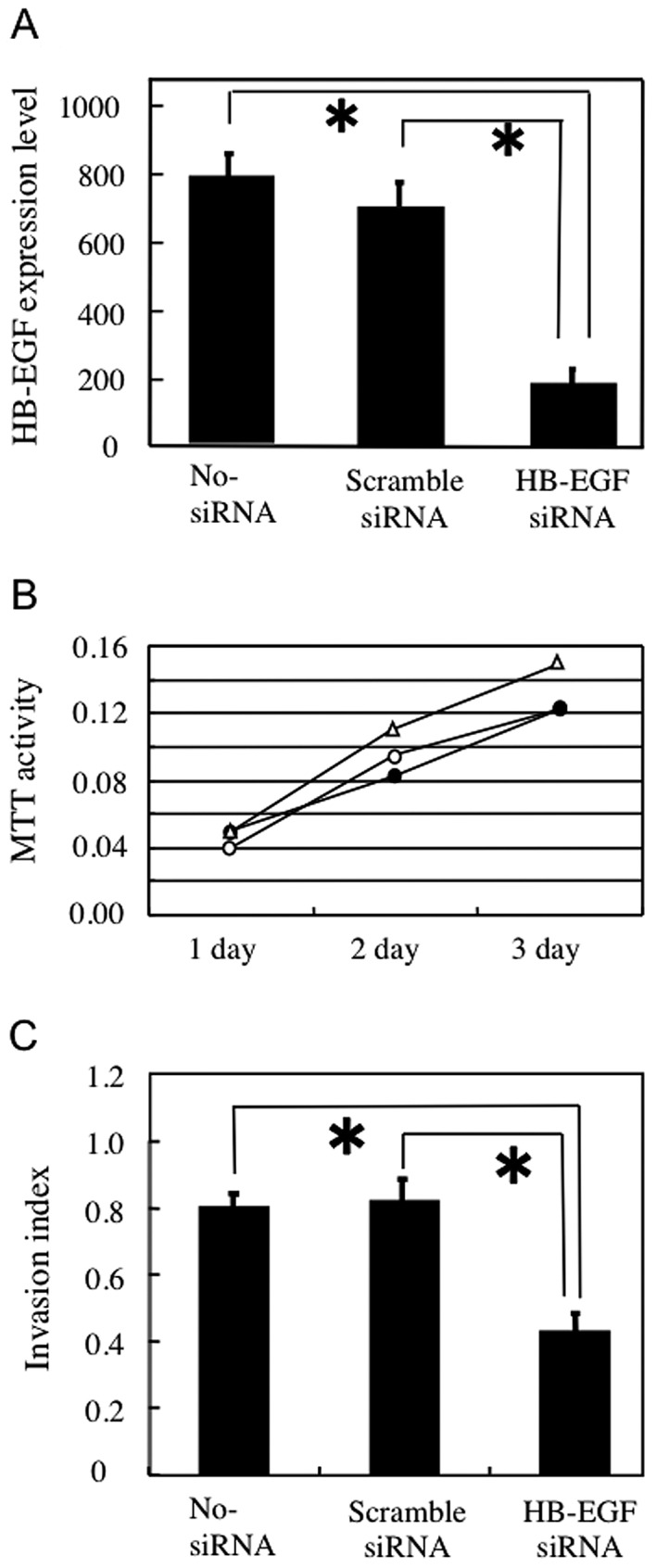
Knockdown of heparin-binding epidermal growth factor-like growth factor (HB-EGF) affects invasion activity in HSC3 cells. (A) Expression of HB-EGF mRNA in HSC3 cells treated with siRNAs was determined by real-time RT-PCR. The amount of HB-EGF mRNA was significantly reduced in HB-EGF siRNA-treated HSC3 cells compared with that in control no-siRNA or scrambled-siRNA-treated HSC3 cells. (B) Effect of HB-EGF on HSC3 cell proliferation. The MTT assay showed similar proliferation rates among cells treated with HB-EGF siRNA (closed circle), no-siRNA (triangle), and scrambled-siRNA (open circle). (C) Invasion activity of HB-EGF siRNA-transfected HSC3 cells as determined by the Matrigel invasion assay. The invasion index represents the ratio of cells migrating through a Matrigel-coated membrane/cells migrating through a control non-coated membrane. No-siRNA and scrambled-siRNA transfectants were highly invasive, whereas the invasiveness of HB-EGF siRNA-transfected HSC3 cells was significantly lower.

To determine the role of HB-EGF in oral SCC cells, we performed HB-EGF knockdown using siRNA in HSC3 cells with high HB-EGF expression. Cells treated with oligofectamine reagent only (no-siRNA) and cells treated with scrambled siRNA were used as controls for non-specific effects. The HB-EGF mRNA expression level did not differ significantly between the scrambled siRNA and no-siRNA transfectants, but HB-EGF mRNA expression was obviously reduced in the HB-EGF siRNA transfectants (Fig. 2A). As HB-EGF has been shown to stimulate growth in a variety of cells (13), the effect of HB-EGF siRNA treatment on HSC3 cell proliferation was analyzed by the MTT assay. After 1, 2 and 3 days of culture, there was no difference in MTT activity between HSC3 cells transfected with HB-EGF siRNA and cells transfected with scrambled siRNA (Fig. 2B). Furthermore, no difference in cell density was observed among the three treatments (data not shown). These data indicate that HB-EGF had no effect on HSC3 cell proliferation.

The effect of HB-EGF siRNA treatment on the invasion potential of HSC3 cells was analyzed by a Matrigel invasion assay. Invasion activity did not differ between the no-siRNA and scrambled siRNA transformants, whereas transfection with HB-EGF siRNA significantly decreased the number of invasive HSC3 cells (Fig. 2C). These results suggest that HB-EGF is associated with the invasion activity of HSC3 cells.

### Secreted HB-EGF is associated with the MMP-9 level in HSC3 cells

**Figure 3:**
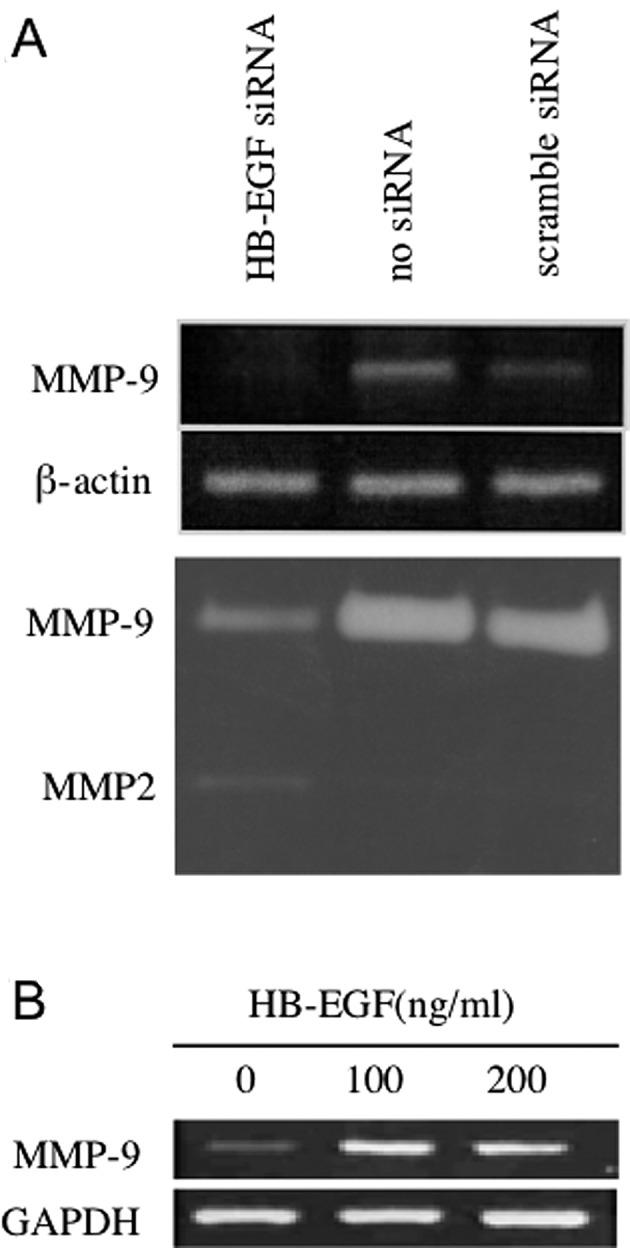
Heparin-binding epidermal growth factor-like growth factor (HB-EGF) upregulates MMP-9 expression. (A) MMP-9 mRNA expression and collagenase activity in siRNA-treated HSC3 cells was determined by RT-PCR and zymography, respectively. The MMP-9 mRNA level was reduced in HB-EGF siRNA-treated cells compared with the levels in control no-siRNA and scrambled-siRNA-treated HSC3 cells. (B) The addition of s-HB-EGF to the culture medium upregulated MMP-9 mRNA levels in HSC3 cells.

Secreted and activated MMPs, including MMP-2 and MMP-9, degrade a variety of extracellular matrix macromolecules and thus facilitate cell invasion (14). The effects of HB-EGF siRNA treatment on the MMP-9 mRNA level and collagenase activity were determined by semi-quantitative RT-PCR and zymography, respectively. The MMP-9 mRNA level and collagenase activity were lower in HSC3 cells treated with HB-EGF siRNA compared with the control cells (Fig. 3A), suggesting that HB-EGF synthesized in HSC3 cells upregulates MMP-9 mRNA expression. To examine whether soluble HB-EGF upregulates MMP-9 mRNA levels in HSC3 cells, we added soluble HB-EGF to the culture medium and assessed the MMP-9 mRNA level by RT-PCR. The added HB-EGF induced MMP-9 mRNA expression (Fig. 3B), indicating that HB-EGF released from HSC3 cells upregulates MMP-9 in HSC3 cells in an autocrine manner.

### HB-EGF is shed and activates EGFR on HSC3 cells

**Figure 4:**
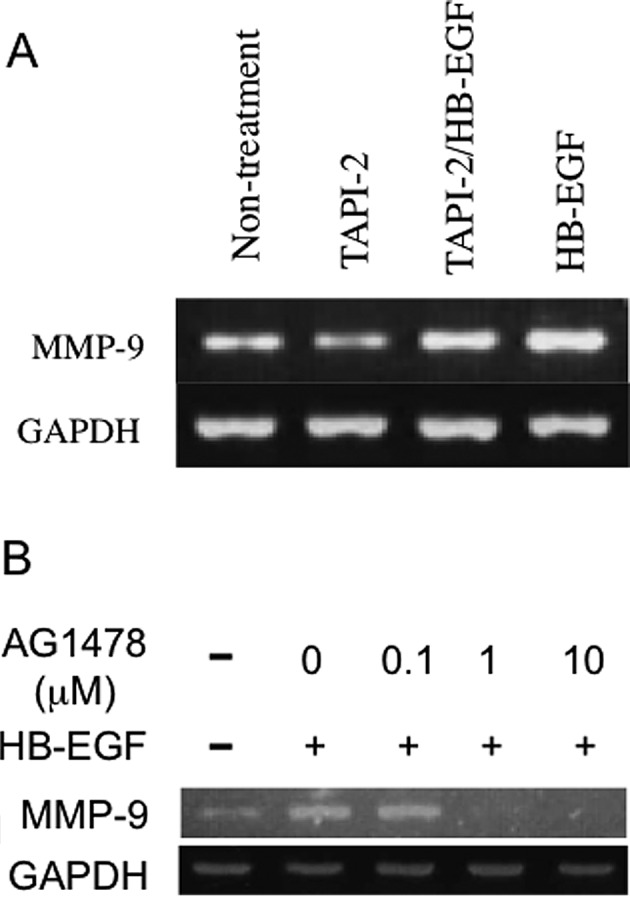
Shedding of HB-EGF upregulates MMP-9 expression through EGFR receptor activation. (A) The TACE inhibitor TAPI-2 reduced MMP-9 mRNA levels in HSC3 cells. (B) The EGFR inhibitor AG1478 downregulated MMP-9 mRNA levels in HSC3 cells

HB-EGF is initially synthesized as a membrane-bound precursor (pro-HB-EGF), which is subsequently cleaved by a membrane sheddase. The cleaved ectodomain plays several roles as a soluble factor (s-HB-EGF). To assess the involvement of the candidate sheddase ADAM17, we analyzed the MMP-9 mRNA level in HSC3 cells treated with an ADAM17 inhibitor, TAPI-2. The MMP-9 mRNA level was decreased in HSC3 cells treated with TAPI-2 (Fig. 4A). Furthermore, the addition of s-HB-EGF to HSC3 cells treated with TAPI-2 resulted in increased MMP-9 mRNA levels (Fig. 4A), showing that TAPI-2 did not act by inhibiting s-HB-EGF. These data suggest that pro-HB-EGF is cleaved by ADAM17 to release s-HB-EGF, which stimulates MMP-9 mRNA upregulation in HSC3 cells.

To examine whether s-HB-EGF upregulates MMP-9 mRNA via EGFR stimulation, we analyzed the effect of the EGFR inhibitor AG1478 on MMP-9 mRNA levels in HSC3 cells. The addition of AG1478 at concentrations greater than 1 μM reduced the basal level of MMP9 mRNA in HSC3 cells as well as the HB-EGF-induced upregulation of MMP-9 mRNA (Fig. 4B). Thus, s-HB-EGF appears to upregulate MMP-9 mRNA expression through the activation of EGFR.

## Discussion

This study provides evidence that HB-EGF acts as an autocrine EGFR ligand to augment cell invasion activity through MMP-9 upregulation in the oral SCC cell line HSC3. Similarly, it has been shown that the invasion ability and MMP-9 levels in ovarian cancer cells are stimulated by HB-EGF expression (7,11). We further showed that the EGFR ligands, AREG, EPGN, EREG and TGF-α as well as HB-EGF are expressed at high levels in HSC3 cells, whereas EGF and BTC are expressed at low levels. Gastrointestinal stromal tumors express several EGFR ligands, but not EGF (15). Although EGF can elicit a variety of biological actions, including the proliferation and differentiation of epithelial and mesenchymal cells (16), particularly in the embryonic stage (17), EGFR ligands other than EGF may play crucial roles in cancer cells.

HB-EGF is initially synthesized as a pro-HB-EGF and is cleaved at the apical plasma membrane to yield s-HB-EGF (18–20), which transactivates EGFR in a variety of cell types (21,22). Ectodomain shedding, the proteolytic processing of the extracellular domain of pro-HB-EGF to release s-HB-EGF, is mediated by several membrane-anchored enzymes, including MMP-2, MMP-3, MMP-9, tumor necrosis factor-α-converting enzyme/a disintegrin and metalloprotease 17 protease (TACE/ADAM17), and ADAM10 (19,20,23–26). In our results, TAPI-2, a specific inhibitor of TACE (27), inhibited HB-EGF activity, indicating that s-HB-EGF, released from the cell membrane by TACE/ADAM17, is necessary for regulation of MMP-9 levels and invasion activity of HSC3 cells. The HB-EGF/EGFR autocrine loop via ADAM17 may play a crucial role in the aggressive behavior of oral SCCs, although further investigation is needed to confirm this.

The standard treatment for oral cancer is a combination of surgery, radiation and chemotherapy. Cytotoxic agents, including carboplatin, cisplatin, and paclitaxel, form the cornerstone of chemotherapy for oral cancer. However, some patients with advanced oral cancer relapse and ultimately die due to the development of drug resistance. Alternative cancer therapies target molecules overexpressed in cancer cells. For example, many human tumors exhibit overexpression of the ErbB family of receptors, including EGFR, which correlates with more aggressive tumors, poor prognosis, and resistance to therapy (28,29). Thus, the ErbB family has become a target of cancer therapy, although the actual curative effects remain insufficient. A large percentage of patients who are initially responsive to ErbB receptor-targeted therapies later become resistant. It is possible that therapy targeted against HB-EGF may overcome such obstacles. It has been suggested that CRM197, a specific inhibitor of HB-EGF, is a promising therapeutic agent for advanced ovarian cancer (30). In addition, the shedding machinery may be a good target for cancer therapy. Inhibition of HB-EGF shedding results in the accumulation of pro-HB-EGF and promotes E-cadherin expression, leading to suppression of EGFR activity. Upregulation of E-cadherin by pro-HB-EGF not only produces cellular morphological changes but also decreases cell motility and enhances apoptotic sensitivity in response to gemcitabine-erlotinib treatment (31). Thus, targeting the cleavage of HB-EGF by ADAM17 may be effective in overcoming chemotherapy resistance in oral cancer.
